# Clinical profile, outcomes and improvement in symptoms and productivity in rhinitic patients in Karachi, Pakistan

**DOI:** 10.1186/1472-6815-9-12

**Published:** 2009-12-11

**Authors:** Taimur Saleem, Umair Khalid, Ubaid Ur Rehman Sherwani, Shehzad Ghaffar

**Affiliations:** 1 Medical College, Aga Khan University, (Stadium Road), Karachi (74800), Pakistan; 2 Ziauddin Medical College, Ziauddin University, (Shahrah-e-Ghalib), Clifton Karachi, Pakistan; 3 Section of Head and Neck Surgery, Department of Surgery, Aga Khan University, (Stadium Road), Karachi (74800), Pakistan

## Abstract

**Background:**

Rhinitis can cause a heavy toll on patients because of its bothersome effects on productivity. This retrospective study was conducted to explore the clinical profile, outcomes and improvement in the symptoms and productivity resulting from treatment of allergic rhinitis in Pakistan.

**Methods:**

We carried out a retrospective file review of all allergic rhinitis patients who presented to the Ear, Nose, Throat Consulting Clinic from January, 2006 to June, 2008 using a structured proforma especially designed for this purpose. Data was entered and analyzed using SPSS v. 16.0.

**Results:**

The charts of 169 patients were reviewed. The mean age of the patients was 35.2 ± 9.1 years. Sixty percent patients were male. Ninety eight patients (58%) reported allergy symptoms to be present at both home and work. One hundred and two patients (60.4%) had symptoms severe enough to cause absence from work or academic activities. Up to seventy one percent patients were spending between 1000 - 3000 Pakistani Rupees (1 US$= 83.3 Pakistani rupees) on the treatment of allergic rhinitis per year. One hundred and fifty one patients (89.3%) reported an improvement in rhinitic symptoms and productivity while 18 patients (10.7%) didn't. This improvement was significantly associated with satisfaction with treatment (p < 0.001).

**Conclusion:**

Allergic rhinitis, a ubiquitous disease, was seen to cause a strain on patients in the form of recurrent treatment-related expenses as well as absenteeism from work or other daily activities. Symptoms and productivity improved significantly after treatment.

## Background

Allergic rhinitis (AR) is characterized by the inflammation of the nasal mucosa. It is induced by exposure to allergens that trigger an immunoglobulin E (IgE) mediated inflammatory response that can result in chronic or recurrent symptoms of rhinorrhea, congestion, and sneezing. The condition can be seasonal or chronic and originates from airborne agents such as pollens, mold spores, and dust-borne mites [1-5].

AR represents a global health issue affecting 10% to 25% of the world population. The increasing prevalence of this condition has resulted in a significant impact on the Quality of Life (QOL) in addition to imposing a considerable socio-economic burden on patients [6,7]. AR is a major chronic respiratory disease by virtue of its high prevalence and significant effect on, work or school performance, and productivity [5]. It also makes asthma control a difficult task for patients and their clinicians and is one of the ten leading reasons for seeking primary health care [1,8].

The determinants of QOL in AR are imperfectly understood at best. More specifically, the influence of socio-demographic factors on QOL in patients with allergic rhinitis has been little investigated so far. In a study done in France, it was reported that residence in the countryside, female gender and a lower education level were independent predictors of poorer QOL in AR patients [9]. Moreover, most of the current literature on allergic rhinitis has focused on developed countries whereas research from developing countries on the subject is not adequately represented.

Improvement in symptoms and productivity in patients with AR has an important relationship with a multitude of clinical, immunological, and functional parameters [9,10]. AR represents a debilitating drain on the health, financial and other resources in the form of multiple clinic visits and multiple days lost from work or school. All of these parameters compositely translate into impeded productivity for a developing country like Pakistan where health care is already plagued by issues of affordability and accessibility. Limited literature is available regarding the management and subsequent improvement in symptoms and productivity after treatment of AR in Pakistan. Our study, therefore, serves as an important document to fill the gaps in information with respect to the local perspective. We aimed to describe the socio-demographic characteristics of patients with AR and to assess the improvement in symptoms and productivity after administration of therapy.

## Methods

### Study design and setting

We carried out a retrospective file review of all AR patients who presented to a single faculty member at the Aga Khan University Hospital (AKUH) Ear Nose Throat (ENT) Consulting Clinic from January, 2006 to June, 2008.

### Inclusion and exclusion criteria

All patients older than 10 years of age and with a clinical diagnosis of AR were selected for this study. AR was diagnosed based on a thorough history and a complete clinical examination by a single faculty member to ensure uniformity in diagnosis. By using this comprehensive clinical approach, patients with AR were diagnosed and distinguished from patients affected by vasomotor, infectious or other inflammatory rhinitis. Similarly, patients with other nasosinusal anomalies presenting with rhinitic symptoms were excluded on the basis of history and physical examination. Patients with associated surgical pathology such as deviated nasal septum (DNS), hypertrophic turbinates or nasal polyposis were scheduled for appropriate surgical procedure and excluded from this retrospective review. Unfortunately, skin testing and specific IgE testing against allergens was not performed because of financial restraints cited by most of the patients attending the clinic. The cost of IgE antibody is PKRs. 1,050 while skin allergy testing costs around PKRs. 1,500. According to recent financial reports, the per capita income in Pakistan has grown over the past few years [11] but is very closely paralleled by increases in inflation, and conducting expensive tests in this clinical arena may not be a financially feasible option for many patients.

### Ethical considerations

The protocol of this study was submitted to the Ethical Review Committee (ERC) at AKUH. Being a retrospective study, the protocol was granted exemption from review as per guidelines of the ERC. Informed consent was not needed owing to the anonymous presentation of the patient data as per guidelines of the ERC at AKUH.

### Management Regime

The type of drug prescribed to the patients for the treatment of AR was random; some patients received more than one medication. Patients were also counseled about the non-pharmacological treatment measures for AR such as steam inhalation and lifestyle changes to avoid exposure to allergens. The mean duration of therapy was 3 months.

### Improvement in symptoms and productivity

A standardized tool for measuring QOL was not used because of two primary reasons:

a) Firstly, this was a retrospective chart review whereby a QOL measuring tool could not be administered at baseline and terminus of treatment.

b) Secondly, the use of QOL tools in a high volume, busy ENT clinic in a developing country is a highly arduous task in actual clinical practice.

Measurement of improvement in quality of life of patients was therefore simplified for all practical purposes and assessed using a composite score based on variables such as reduction of complaints after institution of treatment as well as reduction in absenteeism from work or other daily activities such as school/college. Patients were asked about improvement in symptoms and productivity at the end of treatment and this was documented in the patient's charts. Patients reporting more than 50% improvement across the variables of symptoms and productivity as compared to their baseline situation were categorized as having an improvement.

### Data collection, management and statistical analysis

The data was collected on a pre-tested, structured proforma especially prepared for the purpose. The data procured from the file review were coded and entered in SPSS version 16.0 for analysis. In descriptive analysis, the mean and standard deviation of continuous variables and percentages of categorical variables were computed. Associations were evaluated using chi-square test and Fisher's exact test where applicable. A p-value of < 0.05 was considered statistically significant, unless otherwise specified.

## Results

A total of 169 files were reviewed in accordance with the inclusion criteria used in our study.

### Socio-demographic data

The mean age of the patients was 35.2 ± 9.1 years. One hundred and two patients (60.4%) were male while 67 (39.6%) were female. Most of the patients in the sample were well educated. The nature of the job was primarily office work in 83 patients (49.1%) and primarily field work in 20 patients (11.8%).

### Variables related to allergic rhinitis

The allergens were reported as being household by 120 patients (71%), environmental by 137 patients (81.1%), insect-related by 1 patient (0.6%), animal-related by 13 patients (7.7%) and occurring at place of work by 10 patients (5.9%). Ninety eight patients (58%) reported allergy symptoms to be present at both home and work, 29 patients (17.2%) reported allergy symptoms to be present at work only while 42 patients (24.8%) reported the allergy symptoms to be present at home only. Most of the patients (24.3%) reported a daily exposure time to allergens between 6 to 8 hours; this was based on patient perception of symptoms and allergens. Baseline features of our sample have been shown in table 1. Sneezing (79.9%), watery rhinorrhea (76.9%) and nasal congestion (75.7%) were the most common complaints of the patients at the time of presentation to the ENT Consulting Clinic. In addition, many patients reported ocular complaints (43.8%) and post nasal drip (55%) as well. Additional systemic complaints reported by patients included generalized weakness (79.9%), fatigue (76.9%), difficulty in concentrating (48.5%), irritability (46.2%) and decreased sleep at night (38.5%). Change in climate (89.3%) and exposure to various odors (77.5%) were reported as the most common triggering factors for symptoms by the patients.

**Table 1:** Baseline characteristics of patients with allergic rhinitis

Baseline variables of patients with allergic rhinitis	Frequency (%) (n = 169)
**Age in years**	

10-20	15 (8.9)

21-30	39 (23.1)

31-40	60 (35.5)

41-50	46 (27.2)

51-60	7 (4.1)

61-70	2 (1.2)

**Gender**	

Male	102 (60.4)

Female	67 (39.6)

**Education**	

Primary (till grade 5)	5 (3)

Secondary (till grade 10)	12 (7.1)

Higher secondary (till grade 12)	35 (20.7)

Graduation/post-graduation	117 (69.2)

**Nature of job**	

Office work	83 (49.1)

Field work	20 (11.8)

Both types of work	15 (8.9)

Neither type of work	51 (30.2)

**History of any known allergies**	165 (97.6)

**Family history of allergies**	129 (76.3)

**Place where allergy symptoms are present**	

Home	42 (24.8)

Work	29 (17.2)

Both	98 (58)

**Exposure time to allergens in 24 hours (in hours)**	

0-2	32 (18.9)

> 2-4	18 (10.7)

> 4-6	36 (21.3)

> 6-8	41 (24.3)

> 8-12	31 (18.3)

> 12	2 (1.2)

All the time	9 (5.3)

### Social and financial impact of symptoms

One hundred and two patients (60.4%) had symptoms severe enough to cause absence from work. Eighty nine patients (87.2%) reported at least 1 - 2 days of absence from work while the remaining 13 patients (12.8%) reported missing from 3 to 8 days of work; this absenteeism occurred in association with the presence of symptoms. Up to seventy one percent patients were spending between 1000 - 3000 Pakistani Rupees on the treatment of AR per year in the form of consultation fees and medications costs. Twenty nine patients (17.2%) were spending between 100 - 1000 Rupees while 20 patients (11.8%) were spending more than 3000 Rupees on treatment of AR.

### Treatment of allergic rhinitis

Seventy six patients (45%) were not using any medication before consulting the ENT specialist at AKUH. The remaining patients were either using antihistamines [58 (34.3%)], steroids [9 (5.3%)], analgesics [40 (23.7%)] or antibiotics [24 (14.2%)]. Antihistamines (95.3%) and steroids (79.9%) were the most commonly prescribed medications for the treatment of AR at our institute. In addition, leukotriene receptor antagonist was also prescribed to 69 (40.8%) patients. Less commonly prescribed medications were anticholinergic agents [11 (6.5%)] and sympathomimetic decongestants [57 (33.7%)]. Table 2 shows the trigger for symptoms and the frequency of absence from work in patients with AR.

**Table 2:** Trigger for symptoms and absence from work in patients with Allergic Rhinitis

Variables of patients with allergic rhinitis	Frequency (%) *
**Triggers for symptoms (n = 169)**	

Change in climate	151 (89.3)

Odors **	131 (77.5)

Hot or spicy food	10 (5.9)

Emotional changes	28 (16.6)

Environmental factors^	43 (25.4)

None of the above	7 (4.1)

**Frequency of absence from work (n = 102)**	

Every 1 month	16 (15.7)

Every 2 months	35 (34.3)

Every 3 months	33 (32.3)

Every 4 months	13 (12.7)

Every 5 months	2 (2)

Every 6 months	1 (1)

More than 6 months	2 (2)

* Some percentages don't add upto 100% because of multiple response questions

** Perfumes, cigarette smoke, paint fumes and ink

^Barometric changes, bright lights

### Improvement in symptoms and productivity

On the whole, 151 patients (89.3%) reported an improvement in their symptoms and productivity while 18 patients (10.7%) didn't. The associations between this improvement and various factors have been shown in table 3. One forty nine patients (88.2%) had reported satisfaction with their treatment while the remaining 20 patients (11.8%) were not satisfied with their treatment. Improvement in symptoms and productivity was significantly associated with satisfaction with treatment (p < 0.001).

**Table 3:** Associations between the improvement in symptoms and productivity and different variables

Variables affecting symptoms and productivity of patients	Improvement (f)	
	Yes	No
**Age in years (p = 0.87)**		

≤20	14	1

21-40	88	11

> 40	49	6

**Gender (p = 0.57)**		

Male	91	11

Female	60	7

**Education (p = 0.54)**		

Primary (till grade 5)	5	0

Secondary (till grade 10)	11	1

Higher secondary (till grade 12)	33	2

Graduation/post-graduation	102	15

**Exposure time to allergen in 24 hours (in hours) (p = 0.24)**		

≤2	29	3

> 2-6	51	3

> 6	71	12

**Type of medication prescribed**		

Steroids (oral/nasal) (p = 0.49)	120	15

Antihistamines (p = 0.60)	144	17

Sympathomimetic decongestants (p = 0.84)	50	7

Anticholinergics (0.67)	10	1

Leukotriene receptor antagonists (p = 0.54)	62	7

**Duration for which medication was used (in months) (p = 0.94)**		

≤1	36	5

> 1-3	95	10

> 3-6	18	3

> 6-12	1	0

> 12	1	0

**Regularity in use of medication (p = 0.18)**		

Yes	133	14

No	18	4

**Non-pharmacological advice given to patient (p = 0.71)**		

Yes	148	18

No	3	0

**Compliance of patient in following the advice about lifestyle changes (p = 0.12)**		

Yes	142	15

No	9	3

**Satisfaction with treatment (p < 0.001)**		

Yes	139	10

No	12	8

## Discussion

Although considered a relatively benign condition, AR can exact a heavy toll on patients by virtue of its high prevalence and significant effect on work or school performance and productivity.

Although AR is more often seen in children, the age of diagnosis can be quite variable, as it was diagnosed even in the geriatric age group as seen in our study. Precisely consistent epidemiologic data regarding AR are generally difficult to obtain because various studies have used different definitions of AR. We encountered patients aged 10 years and above in the ENT clinic at our institution. The mean age of diagnosis in our patients was, however, 35.2 years. This is in concordance with another study group that evaluated patients of AR; the mean age of diagnosis being 32 years [12].

In our study, more than half of the patients (69.2%) were educated at least till their graduation or post-graduation. Patients who are better educated are more likely to understand the pathophysiological basis of AR and hence the importance of treatment, avoidance of allergens and prevention of episodes of disease. It has already been proven that lower level of education is an independent predictor of a poorer quality of life in patients with AR [9]. However, we did not find a statistically significant association between the education level and improvement in symptoms and productivity in our patients, probably because of the high baseline education level of the majority of the patients.

We found a slight male predominance in our patients, which is in agreement with other studies reported in literature [6,13]. A French study reported that despite male predominance in the prevalence of AR, the female gender is more strongly associated with a poorer QOL [9]. In our study, a significant association between gender and improvement in symptoms and productivity was not seen. We were expecting to see a significant association because of the differential nature of exposure and time of exposure. Females in our regions mostly spend time indoors as compared to their male counterparts and would therefore be exposed to different allergens

An earlier study investigated the types of allergens associated with AR via allergy testing and found that the percentages of positive reactions to grass, tree, and herb pollens were equally high (30 - 40%). Sensitivity to house dust was present in 44% of the patients but the heterogeneous composition of house dust made it difficult to determine the exact allergenic factors. Animal danders were not found to be of great importance [14]. In contrast, a recent Chinese study has demonstrated that dermatophmoides (47%), herbs (19.7%), tree (18%), animal dander (8.9%) and house dust (6.5%) are important allergens in patients with AR in this order of frequency [15]. In another recent study from the Mersin region, mites and pollen were found to be the most common allergens in patients with AR. This study also concluded that the distribution of allergens in patients with AR is associated with climatic, environmental and socioeconomic factors of the area [16]. Other studies from Konya region and China have shown a similar distribution of allergens [17,18].

In our study, most of the patients reported symptoms in both the indoor and outdoor environments, which suggests that patients were sensitized to various allergens at the same time and the cumulative effect of these exposures was more as compared to isolated exposures. Most of the patients reported a daily exposure time of allergens to be between 6 and 8 hours.

The most common symptoms that were reported by the patients in our study were sneezing, watery rhinorrhea and nasal congestion, which were present in more than half of the patients, but other complaints like post nasal drip, ocular symptoms, itching, cough, sore throat, hoarseness and even decreased sense of taste were also reported. A number of triggers for these symptoms were mentioned by the patients, of which change in climate and odors like perfumes, cigarette smoke, paint fumes and ink, were the main triggers responsible for producing the symptoms. Documentation of these triggers and their subsequent prevention is therefore an important part of patient education and clinicians should be cognizant of this aspect of patient care and disease management.

High incidence of allergic rhinitis, combined with the cold- and flu-like symptoms, may cause absenteeism or reduced productivity while at work when the condition is untreated. In 60.4% of our patients, the symptoms were severe enough to cause absenteeism from work. On further questioning, most patients answered that they missed on average 1-2 days because of their symptoms. Most of these episodes used to occur with a frequency of once every two to three months. Further, many over-the-counter (OTC) treatments for allergic rhinitis have sedative side effects associated with reduction in productivity. Numerous studies have demonstrated a relationship between antihistamine use and subsequent effects on sedation, psychomotor performance, and cognition [19-21]. Hence, the chronic nature of rhinitis, its high prevalence, adverse effects on QOL, and the use of known effective treatments make this a likely target for quality improvement programs [5].

Generally, employers do not consider the potential for productivity losses due to allergic rhinitis to be a major concern. These productivity losses and associated costs may, however, be substantial. Cost issues must be addressed in the management as cost-effective management is fundamental in the proper care of allergic rhinitis. The total cost of allergic rhinitis in the United States was estimated at $7.9 billion in 1997, including $4.5 billion in direct medical costs and an additional $3.4 billion in indirect costs, mostly related to reduced work productivity. AR is responsible for 3.8 million days lost each year from work and school in the United States [3]. In our study, most people reported an expenditure of between Rs. 1000 to Rs. 3000 per annum on medications and consultations regarding their episodes of AR. There are other important aspects in caring for patients with allergic rhinitis that are equally important in achieving the best outcomes for sufferers of allergic rhinitis. Compliance is essential in getting optimal medical management. Issues such as failure to take and improper use of medications as prescribed can lead to dissatisfaction in controlling symptoms in one study [22].

In our study, the patients had been prescribed various medications for the treatment of AR. Most common medication used was anti-histamines, followed by analgesics, antibiotics and steroids. Leukotriene receptor antagonist was also prescribed to some patients. According to our results, none of the medications used seemed to have a significant role in improvement of symptoms and productivity in these patients in comparison to each other. There are two reasons to explain this finding. It could be because many patients were receiving more than one medication at a time; hence the confounding effect of one medication on the effect of the other can't be eliminated. Therefore, it is difficult to comment on which drug exactly had the greatest impact. The retrospective design of the study is also an important consideration in this regard. Secondly, the compliance was measured only in terms of documentation based on subjective parameters, patient statements and recall. Although antihistamines provide temporary relief, studies have shown that intranasal corticosteroids are the most effective medication for reducing congestion in patients with inflammatory nasal conditions [23].

The improvement in symptoms and productivity was significantly associated with satisfaction with treatment (p < 0.001). As mentioned earlier, AR is a condition that significantly impairs the productivity of patients. Improvement in such productivity is one of the primary goals of treatment of this condition and achievement of this goal imparts the patients with a sense of satisfaction from the treatment administered by their physician as was seen in our study.

## Strengths and limitations

This study provides an important perspective regarding the management and outcome of allergic rhinitis from a developing country. However, we would also like to acknowledge the following limitations of this study. Our diagnosis of AR was "sign and symptom" based; this approach was undertaken because of the financial constraints cited by majority of patients who could not afford expensive testing such as skin tests and IgE testing against specific allergens. Also, these tests are not ubiquitously available in Pakistani hospitals. Hence, by using a comprehensive clinical approach, patients with AR were distinguished from patients affected by vasomotor, infectious or other inflammatory rhinitis.

Because of its retrospective nature, a standardized tool for measuring QOL could not be used for this study. Therefore, we used a simplified measure of improvement in terms of symptoms and productivity. This approach appears more practical for a high volume clinic in a developing country like Pakistan where administration of multi-item QOL questionnaires may not be feasible.

Another shortcoming that should be pointed out is the considerable subjectivity involved in measuring the daily exposure time to allergens. This was based on patient's perception and recall of symptoms occurring in that particular setting over a considerable length of time on most days to any known or likely allergen.

## Conclusion

Allergic rhinitis, a ubiquitous disease, has a significant impact on patients because of bothersome symptoms and expenses incurred from treatment as was seen in our study. Current therapy is effective in improving the quality of life in these patients. Current literature on allergic rhinitis from the developing world is lacking and under-represented. Therefore, this study is an important baseline document. The results obtained from our study can help improve outcomes by improving prescribing practices. We acknowledge the limitations of this study, particularly its retrospective design. Prospective studies with more robust designs are needed in our region to correctly document the burden of the disease, to engineer strategies whereby the improvement in symptoms and productivity of patients with AR can be maximized, and to collate the significant findings observed in our study.

## Competing interests

The authors declare that they have no competing interests.

## Authors' contributions

TS conceived the study, collected, entered and analyzed the data and wrote the manuscript. UK entered and analyzed the data along with writing the manuscript. US collected and entered the data and helped in the manuscript writing. SG was involved in study conception and also supervised the project in addition to writing and editing the manuscript. All authors read and approved the final manuscript.

## Pre-publication history

The pre-publication history for this paper can be accessed here:

http://www.biomedcentral.com/1472-6815/9/12/prepub
